# Focal Malonate Injection Into the Internal Capsule of Rats as a Model of Lacunar Stroke

**DOI:** 10.3389/fneur.2018.01072

**Published:** 2018-12-11

**Authors:** Carla Cirillo, Alice Le Friec, Isabelle Frisach, Robert Darmana, Lorenne Robert, Franck Desmoulin, Isabelle Loubinoux

**Affiliations:** Toulouse NeuroImaging Center, Inserm, Université de Toulouse, UPS, Toulouse, France

**Keywords:** stroke, internal capsule, malonate, sensorimotor deficits, brain imaging

## Abstract

**Background:** Stroke is the first cause of disability in adults in western countries. Infarct of the internal capsule (IC) may be related to motor impairment and poor prognosis in stroke patients. Functional deficits due to medium-sized infarcts are difficult to predict, except if the specific site of the lesion is taken into account. None of the few pre-clinical models recapitulating this type of stroke has shown clear, reproducible, and long-lasting sensorimotor deficits. Here, we developed a rat model of lacunar infarction within the IC, key structure of the sensorimotor pathways, by precise injection of malonate.

**Methods:** The mitochondrial toxin malonate was injected during stereotactic surgery into the IC of rat brains. Rats were divided in three groups: two groups received malonate solution at 1.5M (*n* = 12) or at 3M (*n* = 10) and a sham group (*n* = 5) received PBS. Three key motor functions usually evaluated following cerebral lesion in the clinic strength, target reaching, and fine dexterity were assessed in rats by a forelimb grip strength test, a skilled reaching task (staircase) for reaching and dexterity, and single pellet retrieval task. Sensorimotor functions were evaluated by a neurological scale. Live brain imaging, using magnetic resonance (MRI), and post-mortem immunohistochemistry in brain slices were performed to characterize the lesion site after malonate injection.

**Results:** Intracerebral injection of malonate produced a 100% success rate in inducing a lesion in the IC. All rats receiving the toxin, regardless the dose injected, had similar deficits in strength and dexterity of the contralateral forepaw, and showed significant neurological impairment. Additionally, only partial recovery was observed with respect to strength, while no recovery was observed for dexterity and neurological deficit. MRI and immunostaining show volume size and precise location of the lesion in the IC, destruction of axonal structures and Wallerian degeneration of fibers in the area above the injection site.

**Conclusions:** This pre-clinical model of lacunar stroke induces a lesion in the IC with measurable and reproducible sensorimotor deficits, and limited recovery with stabilization of performance 2 weeks post-injury. Future therapies in stroke may be successfully tested in this model.

## Introduction

Ischemic stroke is a devastating disease, being the major cause of acquired disabilities in adults and the second cause of dementia, after Alzheimer disease (1). There is a growing prevalence of strokes affecting the subcortical white matter (WM), especially in elderly people (2). Lesioning of the WM consequent to ischemic stroke causes motor deficits, whose severity strongly depends on the location of the infarct (3). Functional magnetic resonance imaging (MRI) studies in stroke patients have shown that total infarct of the internal capsule (IC) has a negative clinical prognosis and is related to motor impairment. The posterior limb of the IC (PLIC) constitutes the core of the subcortical motor pathway and contains the major corticofugal tract (4–6); thus, damage to this area leads to a persistent motor disability.

To investigate motor deficits and their recovery after stroke, a good pre-clinical model should display damage to the motor pathway. To date, pre-clinical models of stroke mostly rely on large infarctions affecting both white and gray matter. Technical challenges do exist, mainly because of the low WM/gray matter ratio in rodents. In addition, gaining stereotaxic access in order to selectively lesion the IC is made difficult by the elongated, irregular shape of the IC, and its closeness to the ventricle. Among the animal models used to understand the pathophysiology of stroke, only a few studies have investigated subcortical capsular infarcts (7–14). To date, the methods developed include pharmacological, surgical, and photochemical approaches. Endothelin (ET)-1 is a powerful vasoconstrictor and induces reliable and precise focal damage to the IC (11, 12). Despite the wide use of ET-1, this model has a few limitations, i.e., the high mortality rate (14, 15), the only partial destruction of IC fibers and the lack of marked sensorimotor deficits (11). Another pre-clinical model of IC stroke uses electrocoagulation of the anterior choroidal artery (16, 17). This model has limitations related to the delicate surgery. More importantly, it does not always induce volume size-reproducible lesions, because of the variability in vasculature and collateral blood flow, and the motor impairment is not long-lasting (16–18). A third pre-clinical model of lacunar infarction is photothrombosis of the PLIC. This procedure generates circumscribed lesions of the IC by using a special device with relatively low invasiveness and low mortality rate. However, photothrombosis does not allow to control the severity of ischemia in the irradiated area (18) and does not cause durable motor impairment in all injured animals (7).

Here, we developed a pre-clinical model of focal IC lesion by stereotactic injection of malonate into the PLIC of the adult rat brain. Malonate is a competitive inhibitor of the mitochondrial enzyme succinate dehydrogenase of the Krebs cycle and induces energy failure, excitotoxicity and apoptotic cell death in the vascular and parenchymal tissue (19). By causing long-lasting motor impairment in injured animals, this pre-clinical model reproduces the deficits of human ischemic stroke and is valuable for the design of future therapeutic strategies, particularly in the sub-acute and chronic phases of the disease.

## Materials and Methods

### Animals

Thirty-six 12-week old adult female Sprague-Dawley rats (300–350 g, Janvier, Le Genest-St-Isle, France) were used. Animals were maintained and treated according to Council of the European Communities guidelines (Directive of 24 November 1986, 86/609/EEC). This protocol was approved by the Direction départementale de la Protection des Populations de la Haute-Garonne (authorization no. 31125507) and the Comité d'éthique pour l'expérimentation animale Midi-Pyrénées. All efforts were made to minimize the number of animals used and the suffering they experienced.

### Surgical Procedures

**Figure 1:**
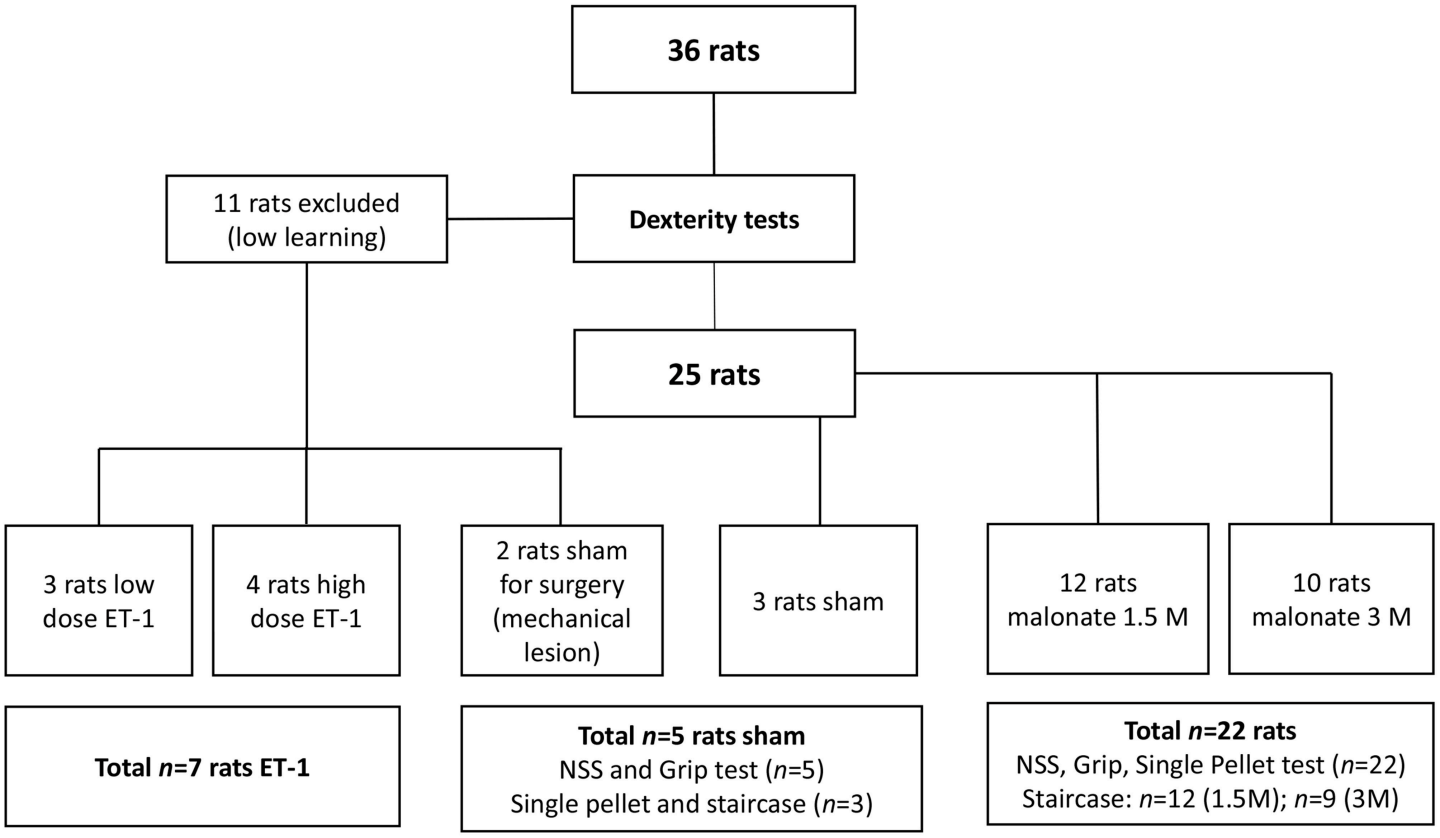
Flow chart of the study. ET, Endothelin; NSS, Neurological severity score.

Rats were divided in four groups: the sham group (*n* = 5) received PBS 0.1%; two groups received malonate solution (pH 7.4 in PBS; Sigma-Aldrich, France) volume 2 μl at 1.5M, (*n* = 12) or 2 μl at 3M (*n* = 10), and the last group (*n* = 7) received ET-1 [volume 1 μl (*n* = 3); volume 1.5 μl or 2.5 μl (*n* = 4) at 0.15 μg/μl) Sigma-Aldrich] (Figure 1). Rats were anesthetized with isoflurane (3% for induction, 2% for maintenance, in 0.7 l/min O2), secured to a stereotaxic frame and pre-medicated with methylprednisolone (0.4 mg/kg). Body temperature, measured by a rectal probe, was maintained at 37°C using a homeothermic blanket. After local intradermal injection of 0.14 ml of lidocaine 2% and scalp incision, a small hole was drilled (1 mm diameter) under saline cooling. Vehicle (PBS), malonate or ET-1 were injected using a 5 μl-syringe (Hamilton 700 Series, Phymep, Paris, France, 250 μm gauge needle) connected to a micro-pump (injection rate: 0.5 μL/min). In pilot experiments on two rats, a large needle 21G (0.8 mm of diameter) was inserted and turned in the IC to see whether a mechanical injury was sufficient to induce major deficits. These two animals did not show any deficit and were assigned to the sham group. The lesioned hemisphere was the dominant one. The stereotaxic coordinates targeted, were: AP −1.5 mm, ML +3.2 mm, DV −5.3 mm (20), as previously determined from primary motor cortically injured rat brain slices with evidence of corticospinal tract degeneration (21). After the injection, the needle was left in place for additional 4 min to avoid backflow. The burr hole was sealed, skin resewn and rats were allowed to recover in their home cages. For the implementation of our protocol, to characterize precisely the location of the injection site at the chosen coordinates, we sacrificed two of the rats that received low-dose ET-1.

### Motor Assessment

The animals were food restricted to 80% of their initial body weight to increase motivation during training and testing. Rats were trained for the dexterity tests 3 times a week for up to 13 weeks, and were evaluated post-lesion 3 times a week for 5 weeks. Eleven of them (30%) were excluded from the behavioral tests due to low learning. In all the tests, the rats learned the same way independently of the group to which they were later assigned, with the exception of two “low learning” rats, who were assigned to the sham group. After the training period, well-performing rats were randomly assigned to the different groups, before executing the surgical procedure (22). Thus, the sham group had *n* = 5 rats (3 had surgery to the right hemisphere), the 1.5M group *n* = 12 (8 had lesion to the right hemisphere), the 3M group *n* = 10 (*n* = 9 for the Staircase, 3 had lesion to the right hemisphere), and the ET-1 group *n* = 7 (Figure 1). Investigators were blinded to the treatment group.

#### Grip Strength Test

All of the rats underwent strength testing (three trials/paw/day) with the grip strength test. To evaluate maximal forelimb muscle grip strength, the animals grasped a horizontal bar of the Grip Strength Meter (Bioseb, France) attached to a dynamometer, and then they were gently pulled away by an experienced handler until their grasp was broken. This test was validated in previous studies by the group (19, 21, 23). The dynamometer measured the maximal isometric force before the animal released the bar. The rats were trained for 3 days to determine a baseline value that was used to calculate the percentage of post-injury performance. This ensured also that the experimenter reproduced the pulling strength reliably (measurement error tolerated up to 10%) and that the animal was sufficiently trained and accustomed to being held. Post-lesion performances of the contralateral forelimb were expressed as a percentage of the pre-lesion baseline value. Each value reported here represents the median ± interquartile range [first quartile (Q1); third quartile (Q3)] of each group.

#### Staircase Test

The staircase test (24) was used to evaluate the reaching capacity and dexterity of the forelimbs (Supplementary Figure 1). The test apparatus consisted of a chamber with a central platform for the rat to climb onto and a set of seven steps on either side. Rats were placed onto the central platform of the apparatus, from which they could pick up small sweet sucrose treats (pellets) (Dustless Precision Sucrose Pellets, Bioserve) placed into the wells of the staircases, on either side. Forelimbs were assessed separately, as the right staircase is reached with the right paw and the left staircase with left paw. The rats remained in the staircase for 10 min and the total number of pellets eaten on each side was recorded. Performance was measured by the number of successfully retrieved reward pellets. This test determined the laterality of each animal, which was then used to determine the dominant hemisphere and, consequently, the lesion site for each rat. After 2.5 months of learning, we determined the baseline (“pre-injury performance”) for each rat in order to calculate the percentage of post-injury deficit with respect to this value. Each value reported here represents the median ± interquartile range.

#### Single Pellet Retrieval Task (SPRT)

This test (25, 26) was used to assess forelimb skilled reaching and precision grip. Animals were placed into a Plexiglas apparatus (45 cm long, 13 cm wide, 40 cm high) and trained to reach a reward pellet with their dominant forelimb through a 1.5 cm gap. At the end of each trial, rats were trained to return to the back of the apparatus so that the pellet could be replaced. The rats were videotaped, and the images used to determine the number of pellets retrieved (“Pellet”). Twenty tests (20 pellets presented one at a time through the slit) were carried out during which the rats were filmed and their score was determined using the following calculation method:

Total success = Pellets/number of trials (20)

#### Neurological Severity Score (NSS)

The NSS comprises three tests to evaluate reflexes, balance, and sensorimotor function (27, 28) (Table 1). The higher the score, the more severe the deficits. Motor function was assessed by the tail raising test, in which an impaired rat will retract its limbs instead of outstretching them when raised by the tail. Sensorial deficits were evaluated using the Proprioceptive Test, in which an impaired rat, when pushed to the edge of a table, will allow its forelimb and/or hind limb to dangle instead of pushing against the experimenters' hand to replace all limbs on the table. In the Beam Balance Test, an impaired rat will not grasp successfully (all four limbs on the surface) a narrow beam.

**Table 1:** Neurological severity score.

**Neurological severity score**	**Points**
**RAISING RAT BY TAIL (MAXIMUM = 3)**	
Flexion of forelimb	1
Flexion of hindlimb	1
Head moved >10° to vertical axis within 30 sec	1
**SENSORIMOTOR TEST (MAXIMUM = 4) LESIONED SIDE**	
Limb placing test: limb stays on the table 0	0
Delay before putting back limb on the Table 1	1
Limb falls down from table	2
**BEAM BALANCE TESTS (MAXIMUM = 6)**	
Balances with steady posture	0
Grasps side of beam	1
Hugs beam and 1 limb fall down from beam	2
Hugs beam and 2 limb fall down from beam, or spins on beam (>60s)	3
Attempts to balance on beam but falls off (>40s)	4
Attempts to balance on beam but falls off (>20s)	5
Falls off; no attempt to balance or hang on the beam (<20s)	6
Total Maximum Points	13

#### *In vivo* MRI

Rats (*n* = 14, group 1.5M *n* = 12 and group 3M *n* = 2) were scanned 6 weeks after injury using a 3T Achieva MRI scanner (Philips). Horizontal and coronal T2-weighted images were obtained (TR: 2600 s, TE: 98 ms, impulsion angle: 90°, FOV: 50 × 39 × 13 mm, matrix: 176 × 150, voxel size: 0.33 × 0.33 × 0.5 reconstructed 0. 31 × 0.31 × 0.5 mm, 13 slices, acquisition time: 36 min). Lesion volume was measured by Region of Interest selection on each slice using MRIcro for each rat of the 1.5M group. These volumes of interest were used to create a color-coded lesion overlap map of injured voxels, in order to provide an overview of all the lesioned brain areas.

### Tissue Processing and Histology

At the end of the study (6 weeks post-lesion), rats were anesthetized with gaseous isoflurane and sacrificed with a lethal intraperitoneal injection of pentobarbital (1 ml). Heparinized 0.1 M phosphate buffer solution (200 ml, 20 min) to wash blood, and then 4% paraformaldehyde solution (200 ml, 40 min) to fix the tissue, were intracardially perfused. The brain was extracted and immersed in sucrose baths of increasing concentration (10, 20, and 30%). Horizontal 80 μm brain sections were cut with a microtome. One in twelve sections was stained with Cresyl Violet acetate according to standard procedure (Nissl staining). For fluorescence immunostaining, additional 250 μm-sections were cut and one in four sections was stained with purified anti-Neurofilament Marker (pan axonal) antibody (clone SMI 312), to investigate the structure of axonal fibers. Briefly, free-floating sections were permeabilized with 0.1% Triton X-100 (Sigma-Aldrich) in PB for 40 min at room temperature, and incubated with blocking solution (3% goat serum). Sections were then allowed to react with primary antibody mouse anti-SMI 312 (1:500, AbCam) for 48 h at 4°C. After PB washes, antibody Alexa Fluor 488 (1:1000, Molecular Probes) was incubated for 24 h at 4°C. For each group, at least three different animals and five sections per animal were analyzed for each method. Images were captured using a fluorescence stereo-microscope (Axiozoom V16; Zeiss).

### Statistical Analysis

The Kruskal-Wallis ANOVA test was used for multiple comparisons. *Post-hoc* comparisons between groups were analyzed using the Mann-Whitney *U*-test. A Bonferroni correction was used to correct for multiple testing.

## Results

### Behavioral Tests

After IC injury by intracerebral injection of malonate (at 1.5M and 3M), rats underwent different behavioral tests to evaluate the post-lesion deficits, compared to rats that had received PBS (sham group). Rats receiving the high dose of ET-1 (*n* = 4) could not be followed because of the 100% mortality rate observed after injection in the IC. Two animals from this group presented excessive saliva secretion suggesting epileptic seizure. On the contrary, rats receiving the low dose of ET-1 (*n* = 3) presented no deficit.

#### Grip Strength Test

**Figure 2:**
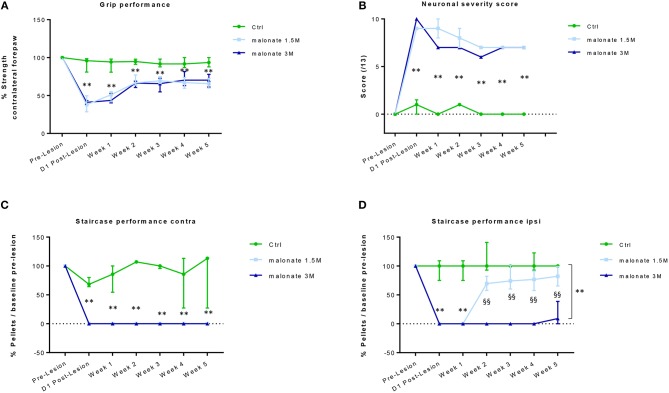
**(A)** The grip strength test of the contralateral forepaw shows that rats receiving malonate at 1.5M (light-blue line) and 3M (dark blue line) had a significant decrease in performance starting at day 1 post-lesion, compared to control rats (green line). Rats receiving malonate showed only partial recovery and stabilization starting at week 2 and lasting till week 5 post-lesion. Results are expressed as a percentage of strength of the contralateral forepaw with respect to pre-lesion values (baseline). Data represent the median values of performance and quartiles. **(B)** Neurological score representing the severity of sensorimotor deficits in rats after malonate injection. The curves show a significantly higher neurological score (values between 0 and 13) in rats receiving malonate at 1.5 M (light blue line) and 3 M (dark blue line), compared to control rats (green line). Data represent the median values and quartiles. **(C,D)** The single pellet retrieval task of the contralateral forepaw **(C)** shows that rats receiving malonate at 1.5 M (light-blue line) and 3 M (dark blue line) had a complete drop to zero in performance starting at day 1 post-lesion, compared to control rats (green line). No recovery of dexterity was observed during the 5 weeks. The single pellet retrieval task of the ipsilateral forepaw **(D)** shows that rats receiving malonate at 1.5 M (light-blue line) and 3 M (dark blue line) had a significant decrease in performance, with rats receiving 3 M dose showing a drop to zero, starting at day 1 post-lesion, compared to control rats (green line). Rats receiving malonate at 1.5 M (light blue line) showed only partial recovery and stabilization starting at week 2 and lasting till week 5 post-lesion, compared to rats receiving 3 M (dark blue line), which did not recover at all. Results are expressed as a percentage of pellets with respect to pre-lesion values (baseline). Data represent the median values of performance and quartiles. **p < 0.05 vs. control rats; ^§§^*p* < 0.05 vs. 1.5 M.

This test allows the measure of forelimb grip strength. The training period ended when minimal variability was obtained between measurements on the same paw and when similar values were obtained on both paws. In the ipsilateral forepaw, a slight decrease in performance was observed immediately after the injury. However, this effect did not reach statistical significance, and was transient, because all rats fully recovered and showed comparable performance over time regardless of the group. A significant effect of the time factor for the contralateral forepaw was observed in all injured rats (Kruskal-Wallis Anova, *p* < 0.005 at each time point), since they showed partial recovery of grip strength over the time, compared to day-1 post lesion (Figure 2A). At day-1 post-lesion, as expected, the analysis revealed a significant difference in the performance of the rats that received malonate (1.5 or 3 M) compared to control animals (sham group, *p* = 0.002 and *p* = 0.004, respectively). Rats receiving malonate at 1.5 or 3 M displayed comparable post-lesion performance, 37.9 and 41.1% of pre-lesion values, respectively, showing no significant effect of the malonate dose on the strength measure. Interestingly, 5 weeks after the injury, the analysis revealed a significant difference between the rats receiving malonate regardless of dose (1.5 or 3M), compared to the control group (*p* = 0.0003 and *p* = 0.01, respectively). However, there was no significant difference between doses of malonate at any time point. Rats receiving malonate only partially recovered after 5 weeks post-injury, with a median performance of 66.4% compared to the ipsilateral paw (IQ [61.7–74%]).

#### Neurological Severity Score

We used a neurological scale to evaluate the severity of the injury after malonate injection. The score varies between 0 and 13 points (a score of 13 points indicates a very severe neurological deficit) (Table 1 and Figure 2B). The statistical analysis revealed that the neurological scores in injured rats receiving malonate at 1.5 or 3 M were significantly higher compared to control rats at all time points (*p* = 0.002 and *p* = 0.004, respectively, Bonferroni corrected, Figure 2B). Comparison between the two injured groups (malonate at 1.5 vs. 3 M) revealed no significant effect associated with malonate dose (Figure 2B).

#### Staircase Test

This test allows the evaluation of sensorimotor function, and in particular of forelimb dexterity, with a focus on the accuracy of reaching and grip using the forepaw (Supplementary Figure 1). This test was used to determine the dominant forepaw of trained rats based on the number of pellets (sweet treats) consumed during the 10 min testing period. All rats were able to seize and eat at least 10 of the 21 pellets with their dominant forepaw. Learning was challenging since it took 2 months to reach a plateau (confirmed, for each animal, by two further weeks of stable performance). Figure 2C shows that the performance of the forepaw of rats receiving malonate (1.5 or 3 M) fell to zero after the injury and did not change over the time, regardless of the dose of malonate, compared to control rats (Figure 2C). After injury, rats did not show recovery of contralateral forepaw dexterity. Though able to reach toward the pellet at the bottom of the wells, they were unable to feel or grasp it during the 5 weeks of observation. Regarding the ipsilateral forepaw, at day-1 and week-1 post-injury, most of the injured rats (malonate at 1.5 and 3 M) scored zero or had much lower performance than the control group (Kruskal-Wallis Anova *p* = 0.006 at both time points, Figure 2D). Then, only the group receiving lower dose of malonate partially recovered. Both groups were significantly different from week 2 to week 5 (*p* = 0.002, all time points) but the 1.5 M group was not different to controls (Figure 2D). At week 5, the median performance was 9.1 and 82.2% for the 1.5 and 3 M groups, respectively. Additionally, it seems that our model of stroke is characterized by a stabilization of the performances at 2 weeks post-injury for the malonate groups at the level of the ipsilateral forepaw (Figure 2D).

#### Single Pellet Retrieval Task

The test allows the evaluation of dexterity in rats and is considered an easier task than the staircase test, as the pellets are placed on a shelf and not at the bottom of a well. None of the rats receiving malonate (1.5 or 3 M) were able to retrieve a pellet after the lesion even after 5 weeks of post-injury evaluation. Only a few rats attempted to seize the pellet on the shelf, but they remained clumsy.

### Histology

**Figure 3:**
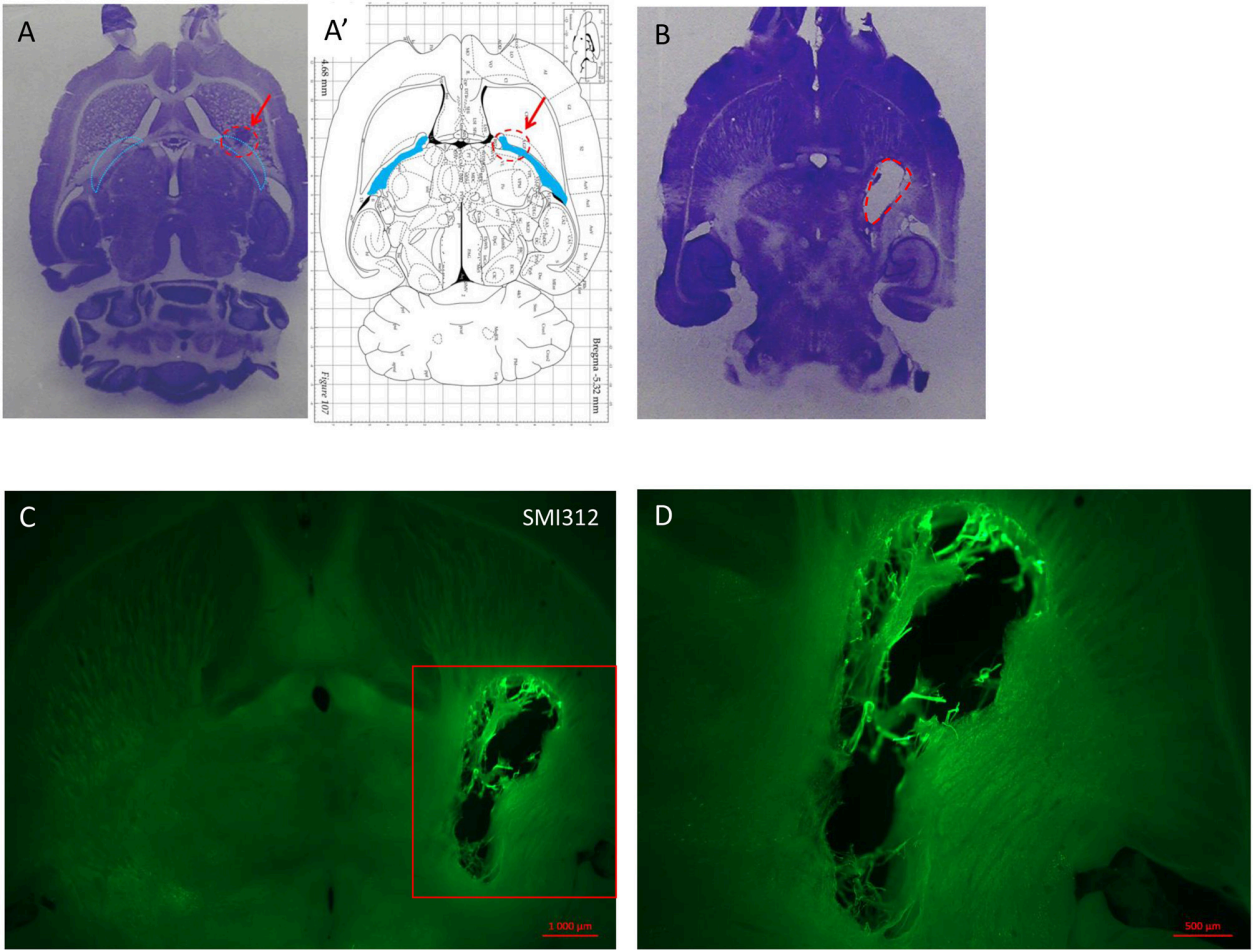
**(A)** Nissl staining on horizontal section of lesioned brain injected with a low dose (1 μl) of endothelin inducing no deficit but showing the injection site in a rat sacrificed 1 week after the injection. **(A′)** Horizontal diagram of the same section on the Rat brain from Paxinos Atlas. The red arrows and the red dashed line in **(A,A′)** indicate the injection site of malonate in the IC (blue lines in **A** and blue area in **A′**). **(B)** Horizontal section of rat brain stained with Nissl showing the lesion volume after 1.5 M malonate injection (dashed red line). The rat was sacrificed 6 weeks after the toxin injection. **(C)** Immunostaining with the axonal marker SMI 312 shows the lesion volume and the destruction of nerve structures in the site of injection (scale bar: 10,00μm). Magnification from the red insert in **(C)** around the lesioned area is shown in **(D)** (scale bar: 500μm).

**Figure 4:**
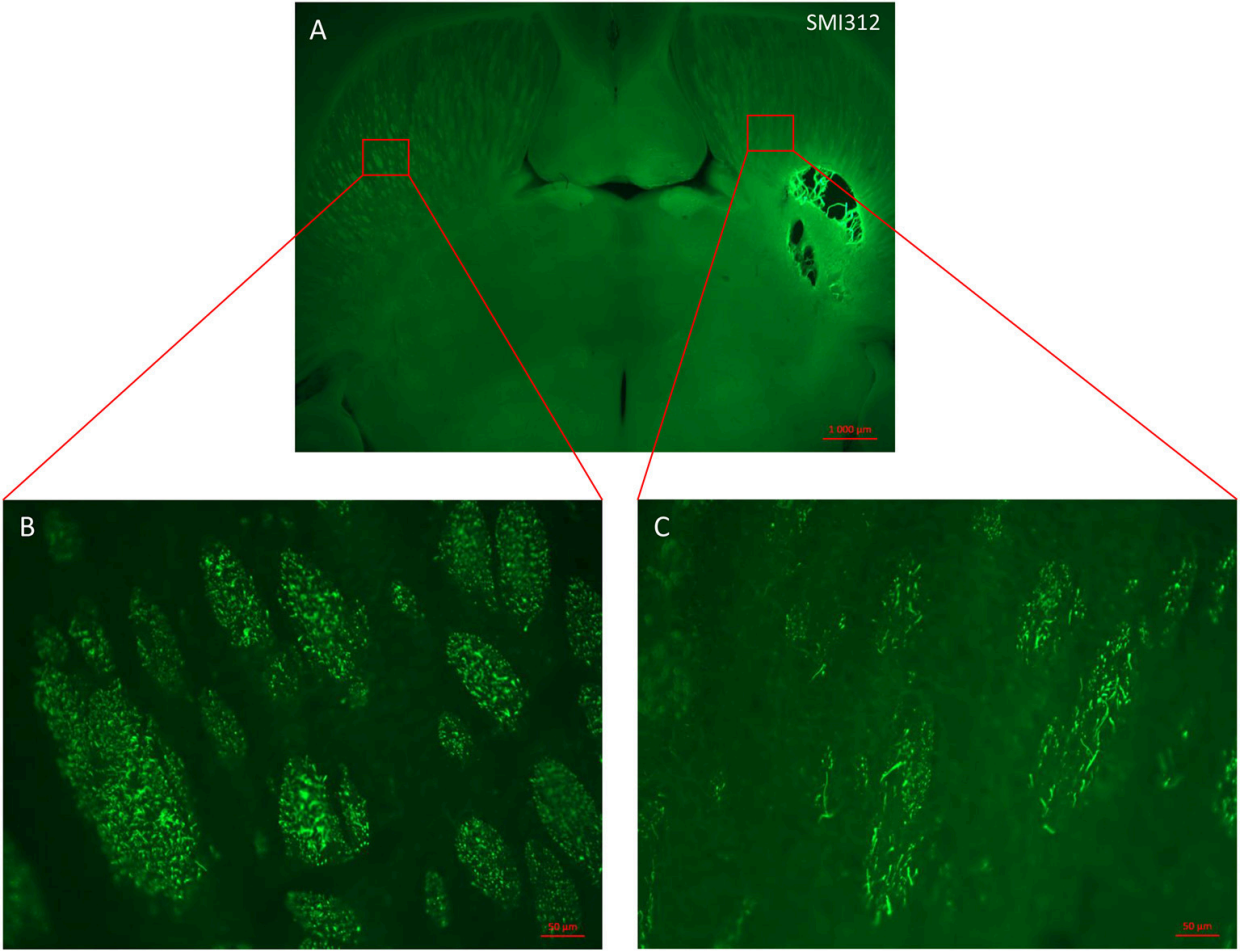
Horizontal section of lesioned rat brain stained with axonal marker SMI 312. **(A)** Malonate injection in the IC caused degeneration of sensorimotor fibers above the injured site (right side scale bar: 1000 μm). **(B,C)** magnification from the red inserts in **(A)** of the control **(B)** and injured **(C)** hemispheres showing the reduced presence of SMI 312-positive nerve fibers (scale bars: 50 μm).

In order to characterize the brain lesion and to evaluate fiber integrity/loss in the damaged area, we performed Nissl staining (Figure 3A). Immunostaining for the neurofilament protein SMI 312 was performed to visualize the structure of axonal fibers within the lesion. Figure 3 confirms that the injection site corresponds to the previously defined location in the IC, and that stereotaxic injection of 1.5 or 3 M of malonate solution produced a unilateral focal forebrain lesion. Histological examination showed circumscribed destruction of the WM area in the malonate model (Figures 3B–D). Absence of fiber staining in the IC also demonstrates the degeneration of the corticospinal tract. Staining with SMI marker allowed the identification of the damaged axonal structures and confirmed neuronal loss (Figure 4). In our model, the lesion in the IC caused degeneration of sensorimotor fibers above the injured site, in the ipsilateral striatum (Figures 4A,C), compared to contralateral side (Figures 4A,B). Below the IC, some degree of lesion to the thalamus was also observed.

### *In-vivo* MRI

**Figure 5:**
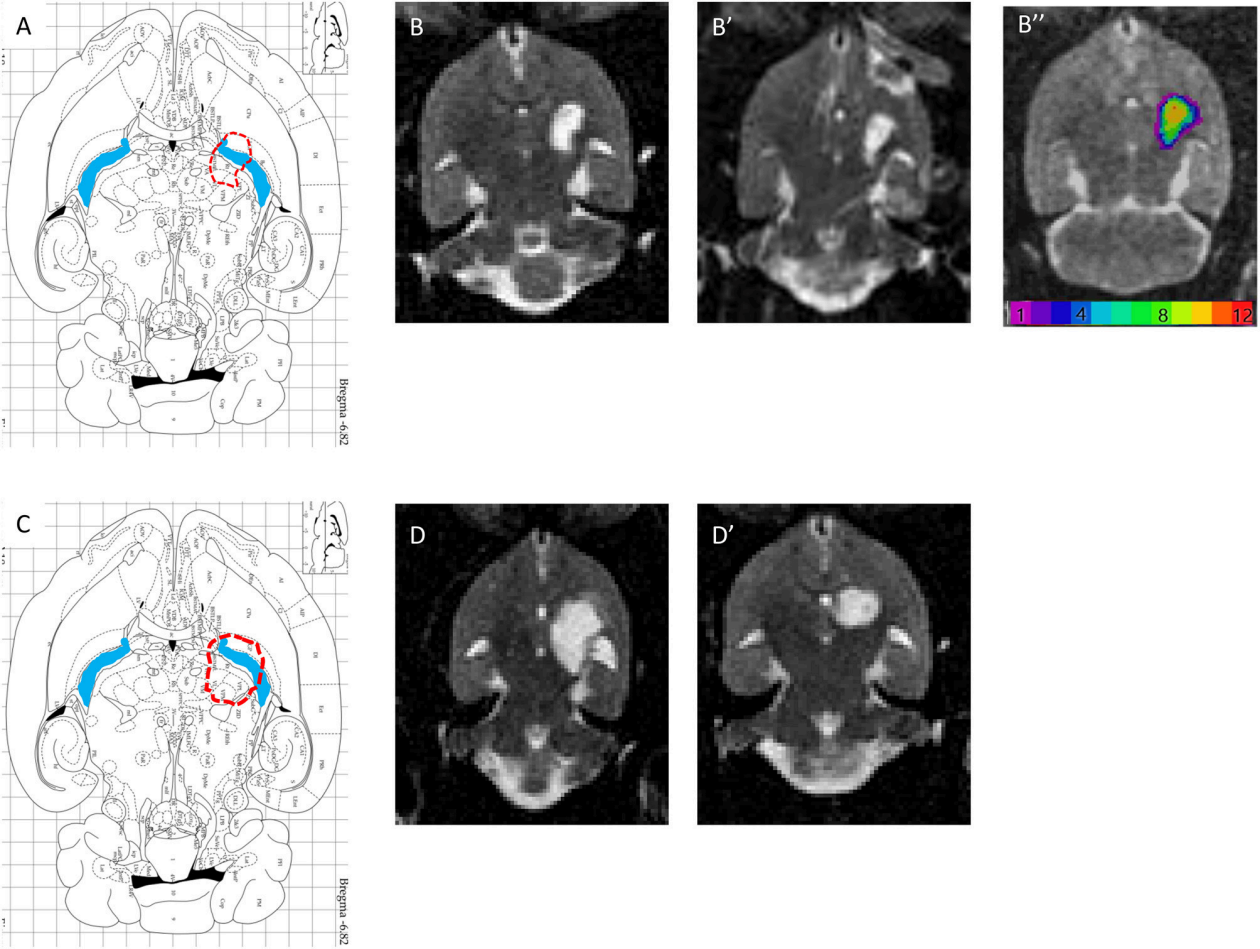
**(A,C)** Horizontal diagram of the brain section on the Paxinos Atlas representing the injection site (red dashed line) and the internal capsule (blue area) in Bregma −6.82 mm. **(B,B′)** Representations of lesion volume in T2 MRI. Horizontal brain section obtained in MRI (T2, reconstructed voxel size: 0.31 × 0.31 × 0.5 mm, −6.8 and −7.3 mm) 6 weeks after injury with 1.5 M malonate. ROI *n* = 293 voxels, or 14.1 mm^3^. **(B″)** color-coded lesion overlap map of injured voxels, providing an overview of all the lesioned brain areas after injection of 1.5 M malonate. **(D,D′)** Representation of lesion volume in T2 MRI. Horizontal brain section obtained in MRI (T2, reconstructed voxel size: 0.31 × 0.31 × 0.5 mm, −6.8 and −7.3 mm) 6 weeks after injection of 3 M malonate. ROI *n* = 509 voxels, or 24.5 mm^3^.

T2 postmortem MRI performed 3 months after the lesion allowed us to measure and assess the variability of lesion volumes (Figures 5B,B′,D,D′) in the injured 1.5 M group (*n* = 12) and in the 3 M group (2 out of 10 animals only). The MRI shows that the lesion site (hyperintense area compared to the opposite side in Figures 5B,B′,D,D′) corresponds to a large area (Figure 5A,C, blue areas). In addition, the adjacent striatum and thalamus were partially lesioned. For the 1.5 M group, the lesion site was centered at −6.3 mm ventral to bregma. Atrophy led to a dilation of the lateral ventricle (the ipsilateral LV was 2.6 times larger than the contralateral LV). The hypertrophied ventricle was included in the final lesion volume. The median lesion volume was 27.8 mm^3^ (first quartile Q1: 22.3; third quartile Q3: 31.2 mm^3^, min = 5.1, max = 53.3 mm^3^). To estimate subcortical damage, striatal and thalamic ROIs were drawn. The lesion comprised 43.3% of striatum and 56.7% of thalamus (median values). No correlation was found between lesion volume and the sensorimotor impairment either at day 1 or at week 5 post-lesion.

## Discussion

In this study, we have established a pre-clinical model of stroke inducing a focal lesion targeting the IC, with long-lasting motor deficits consequent to corticospinal tract damage. Sensory deficits were also observed in this model, because of the destruction of the ascending sensory fibers passing through the IC. Compared to existing pre-clinical models of IC stroke (7, 11, 13, 15, 16, 29), our model led to chronic and measurable sensorimotor deficits. Based on previous studies by our group (19, 23, 30) we used the mitochondrial toxin malonate, a competitive inhibitor of the mitochondrial enzyme succinate dehydrogenase of the Krebs cycle. Malonate was injected by stereotactic surgery into the PLIC, a core structure of the subcortical motor pathway, in the rat brain. In order to validate our model, we first determined the stereotaxic coordinates that would allow precise delivery of toxin to the IC, and the appropriate dose of malonate needed to produce a focal lesion. Second, we evaluated the sensorimotor performance of injured rats using behavioral tests, for which the animals had been previously trained.

In recent studies, we used malonate to cause cortical lesions in the rat brain (19, 21, 30), inducing extensive destruction of the forelimb primary motor cortex (31). Here, we improved this method to target specifically the IC, in order to provide a pre-clinical model for human capsular infarct. So far, animal models of capsular infarct are limited by the debilitating surgery and the important mortality rate, high spontaneous recovery and/or absence of chronic motor deficit and low reproducibility of lesion volume. The novelty of our model resides in the fact that the procedure is safe, cost effective and easy to perform, and the sensorimotor deficits induced by malonate are reproducible in terms of severity and duration. Our model is thus appropriate for studies of sub-acute and long-term injury, although it may be less suitable for studies in the acute phase, as it does not reproduce vascular occlusion. Interestingly, the dose of malonate injected in the IC (1.5 or 3 M) was not a determining factor for the severity of motor deficits. Indeed, the main WM tracts involved in the control of grip strength and dexterity seem to be injured by a circumscribed lesion centered on the PLIC.

In this study, three key motor functions that are assessed as standard in the clinic (strength, target reaching, and fine dexterity), were evaluated in rats using a forepaw grip strength test, single pellet retrieval task (SPRT) and a skilled reaching task (staircase) (19, 21, 26). The grip strength test showed that only the contralateral forepaw of the rats receiving malonate was deficient, compared to control rats, as evidenced by a significant decrease in performance (~60% deficit for all injured groups). No significant difference was observed between the rats receiving 1.5 or 3 M of malonate, indicating that the deficit is not dependent on malonate dose. Five weeks after the injury, all rats displayed a partial recovery of contralateral forepaw grip strength, compared to the initial deficit. However, the impairment remained severe compared to control rats (~25% deficit). In studies by our team (19, 21, 23), we showed that 3 months after the induction of an extensive primary motor cortex (M1) lesion by malonate, rats gradually reached near-total recovery of strength, revealing the presence of compensatory mechanisms. Two IC lesion studies have shown that the extent of the IC lesion (partial or complete) and the target regions (pyramidal tract fibers) are determining factors for the severity and duration of motor deficits (7, 29). In the current study, we show that a small lesion of the IC with the 1.5 M dose of malonate induced long lasting deficits in forepaw strength, and the spontaneous recovery did not reach basal values. This may be due to the fact that, in our model, as shown by MRI and histological data, other fibers such as the striato-thalamic and thalamo-cortical loops are lesioned. This leaves little possibility for compensation of grip strength control by these secondary pathways. One may argue that a limitation of the strength test is that it can be experimenter-dependent. However, the team's experience in the field of stroke (19, 21, 23), together with the strong correlation with the neuronal severity score, demonstrates the robustness of the test.

Regarding the dexterity of the contralateral forepaw, capsular infarct caused significant deficit in rats immediately after injury and, interestingly, regardless of the dose of malonate used. More drastically than strength deficit, no spontaneous functional recovery of dexterity was observed in injured rats for either dexterity test. It seems that major deficits are generated by malonate injection in the IC and that the circuits involved in the compensation are not effective enough to generate even a partial functional recovery of dexterity. It is also possible that the performance could be affected by the configuration of the Staircase Test. In the device, rats are standing on a platform in order to seize the pellets located on either side of the stairs. Injured rats have a less stable posture, limiting their access to the platform, and this decreases their motivation to complete the test. The Single Pellet Retrieving Task seemed easier, but also proved to be too difficult for the animals. Dexterity is a quite difficult and non-ecological task for rodents, and its evaluation requires dietary restriction and considerable learning-time.

Interestingly, we observed that in the Staircase Test the dose of malonate differently affected dexterity of the ipsilateral forepaw in injured rats, by means of more severe chronic deficit observed with the 3 M dose compared to the 1.5 M dose. It is worth mentioning that the cerebral substrates of sensitivity, strength and dexterity are bilateral: the corticospinal axons on the side of the injured hemisphere cross the midline plane and project at the level of the spinal cord to the contralateral side. However, about 10% of direct axons do not cross and project directly to the ipsilateral side (the supposed “healthy” side) (32, 33). This can also be explained by analyzing the MRI images, which show that the 3 M dose induced a larger lesion than the lower dose, suggesting more extensive tissue destruction. In particular, the lesion extended further into the anterior thalamus, known to receive projections from the contralateral motor cortex (34). Stroke patients with lesion in the IC may experience sensory and motor deficits not only in the contralateral, but also in the ipsilateral hand (35–37). However, our previous observations on the cortical lesion model (19, 23) showed that no ipsilateral chronic deficit occurred with such a lesion. Thus, sub-cortical areas and trans-hemispheric cortico-thalamic connections are undoubtedly essential for the control of dexterity by the contralateral side.

In our study, confirmation of the localization of the lesion into the IC and the extension of the damage to the surrounding regions were obtained by *in vivo* brain imaging (MRI) and post-mortem immunostaining. The MRI data suggests a difference in the size of the lesion between the 3 M and the 1.5 M dose of malonate, which also correlates with higher ipsilateral sensorimotor deficit observed in the group of rats receiving the higher dose of the toxin. The staining for neuronal structures shows that the injection site corresponded to IC location and that axonal structures were destroyed in the IC, with the degeneration of the sensorimotor fibers in the M1 area.

In conclusion, our study demonstrates that the malonate lesion to the IC was straightforward and easy to replicate. More importantly, the sensorimotor deficits produced were reproducible with little variability between individuals, and were stable after 2 weeks, with limited recovery after 5 weeks. The presence of long-lasting sensorimotor deficits is consistent with the clinical data reporting loss of strength and dexterity after lacunar infarct in humans.

## Author Contributions

CC and AL: study design, analysis, and interpretation of the data, draft and editing of the manuscript. IF: experimental work and analysis and interpretation of the data. RD, FD, and LR: experimental work. IL: study conception and design, analysis, and interpretation of the data, draft, and editing of the manuscript, obtaining funding. The authors have all read and approved the manuscript.

### Conflict of Interest Statement

The authors declare that the research was conducted in the absence of any commercial or financial relationships that could be construed as a potential conflict of interest.

## Supplementary Material

Supplementary Figure 1 Evaluation of dexterity of the contralateral forepaw of rats during the training period (11 weeks) using the Staircase Test. Number of sugar pellets consumed by using the side of the forepaw who will be subsequently injured according to the group in which the rats will be assigned after. Data represent the median values of performance and quartiles. During the weeks of the training, the number of pellets consumed did increase significantly in all rats, independently of the group in which they were assigned, for the contralateral forepaw [Kruskal-Wallis: H (10, *N* = 275) = 166.2; *p* < 0.001]. This means that the rats reached an increase in their performance over time and had learnt the task well. Statistical analysis of the performance of the contralateral forepaw revealed a significant difference in performance at 3, 4, and 5 weeks of training [Kruskal-Wallis week 3: H (4, *N* = 25) = 11.37; *p* = 0.022]. However, comparative analysis with the Mann-Whitney *U*-Test showed a significant difference between the 1.5 and 3 M malonate groups [Mann-Whitney week 3 *U*-test: *U* = 5; *p* = 0.011], which after correction of Bonferroni was not significant (corrected *p* = 0.066) (the same thing was done for every week and *p* > 0.05).
