# Supplementary figures and images for: Focal Malonate Injection Into the Internal Capsule of Rats as a Model of Lacunar Stroke
Source: Front Neurol. 2018 Dec 11;9:1072. doi: 10.3389/fneur.2018.01072 (PMC6297868; doi:10.3389/fneur.2018.01072)

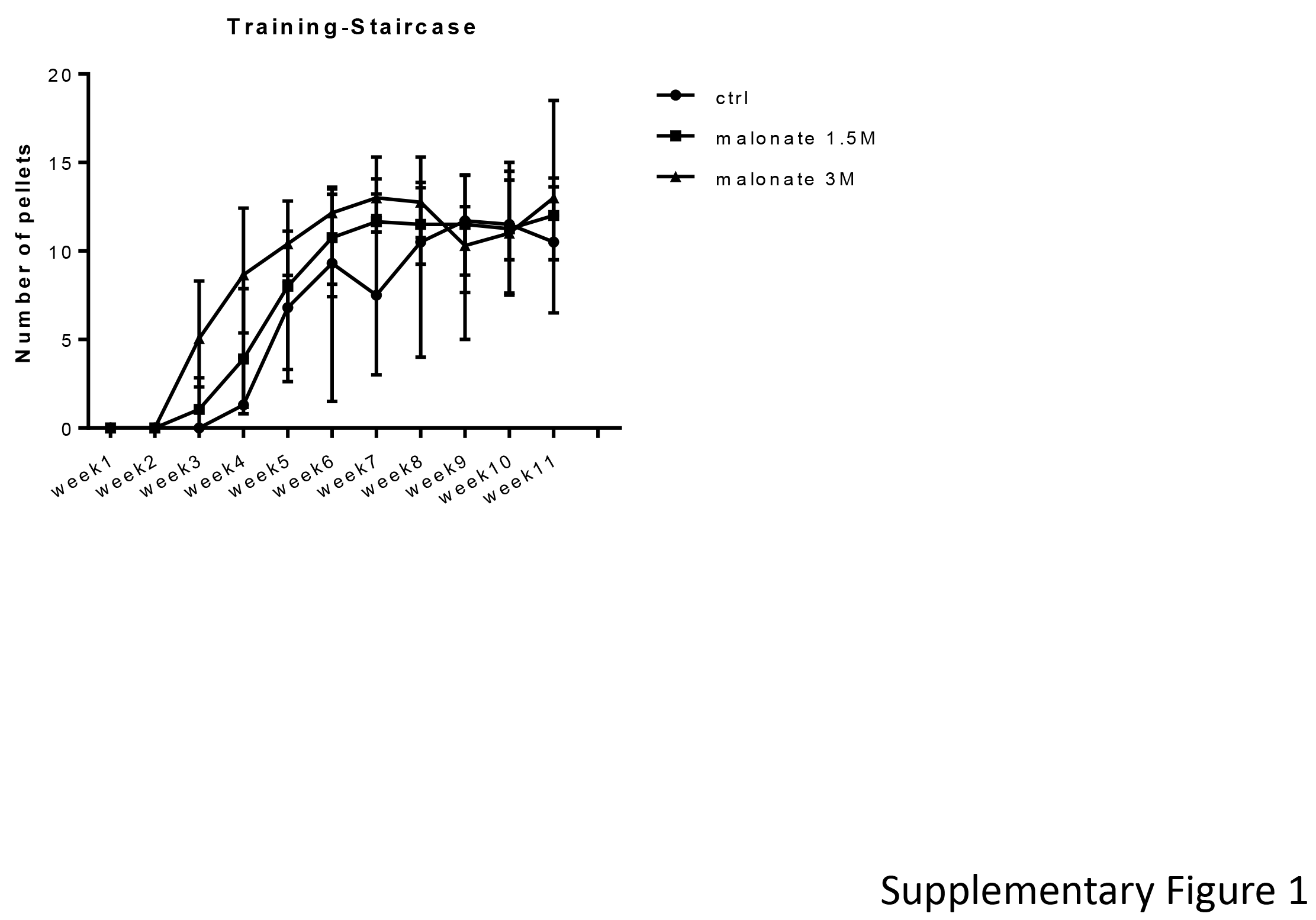

Supplement: Supplementary Figure 1 — Evaluation of dexterity of the contralateral forepaw of rats during the training period (11 weeks) using the Staircase Test. Number of sugar pellets consumed by using the side of the forepaw who will be subsequently injured according to the group in which the rats will be assigned after. Data represent the median values of performance and quartiles. During the weeks of the training, the number of pellets consumed did increase significantly in all rats, independently of the group in which they were assigned, for the contralateral forepaw [Kruskal-Wallis: H (10, N = 275) = 166.2; p < 0.001]. This means that the rats reached an increase in their performance over time and had learnt the task well. Statistical analysis of the performance of the contralateral forepaw revealed a significant difference in performance at 3, 4, and 5 weeks of training [Kruskal-Wallis week 3: H (4, N = 25) = 11.37; p = 0.022]. However, comparative analysis with the Mann-Whitney U-Test showed a significant difference between the 1.5 and 3 M malonate groups [Mann-Whitney week 3 U-test: U = 5; p = 0.011], which after correction of Bonferroni was not significant (corrected p = 0.066) (the same thing was done for every week and p > 0.05). [file Image_1.TIF]
